# Safety of Trypan Blue Capsule Staining to Corneal Endothelium in Patients with Diabetic Retinopathy

**DOI:** 10.1155/2019/4018739

**Published:** 2019-03-27

**Authors:** Hazem Abdelmotaal, Khaled Abdelazeem, Mohamed S. Hussein, Ahmed F. Omar, Walid Ibrahim

**Affiliations:** Department of Ophthalmology, Faculty of Medicine, Assiut University, Assiut, Egypt

## Abstract

**Purpose:**

To study the potential corneal endothelial cell toxicity of trypan blue (TB) when used for phacoemulsification to stain the anterior capsule in patients with diabetic retinopathy.

**Methods:**

This was a single-center, prospective, randomized, individual cohort study. One eye in each patient with diabetic retinopathy underwent phacoemulsification without trypan blue capsule staining (control eye), while the other eye underwent phacoemulsification with trypan blue capsule staining (study eye). Both eyes underwent intraocular lens implantation. Preoperative and four-week postoperative quantitative and qualitative morphometric endothelial cell analyses of the cornea were performed using noncontact specular microscopy.

**Results:**

There were no significant differences in endothelial cell density (mean ± SD for the study group: 2506.74 ± 413.99 cells/mm^2^; mean ± SD for the control eyes: 2466.34 ± 369.12 cells/mm^2^; *P*=0.316) or endothelial cell density (CD) loss% (mean CD loss% was 7.23 ± 13.31 for the study eyes and 9.94 ± 9.36 for the control eyes; *P*=0.157) four weeks after the operation. Additionally, no significant differences were seen in the percentage of hexagonal cells, coefficient of variation, or corneal thickness between the two groups preoperatively and 4 weeks postoperatively.

**Conclusions:**

Direct administration of trypan blue into the anterior chamber for staining of the anterior capsule during cataract surgery did not result in any significant corneal endothelial changes on specular microscopy in patients with severe nonproliferative diabetic retinopathy or high-risk proliferative diabetic retinopathy at 4 weeks postoperatively. This trial is registered with NCT03755752.

## 1. Introduction

Trypan blue (TB) dye staining of the anterior capsule during phacoemulsification allows for successful completion of capsulorhexis when the red reflex is absent or insufficient [1–6].

A diabetic cornea suffers from endothelial cellular dysfunction and dysfunctional repair mechanisms [7, 8]. Furthermore, cataract surgery with phacoemulsification and lens implantation leads to greater degrees of endothelial cell loss in diabetic corneas due to this increased vulnerability to stress and trauma, resulting in greater morphological abnormalities and longer recovery time periods in these patients [9].

Few reports have evaluated the potential cytotoxicity of TB to the corneal endothelium when used for staining the anterior capsule during phacoemulsification [1–3, 6]. Published studies have also not yet established the safety of TB for use on the corneal endothelium of diabetic patients, which is often more vulnerable to damage than that of healthy individuals.

The present clinical study was conducted to evaluate the possible impact of TB on the corneal endothelium of diabetic patients over a four-week-long follow-up period using a contralateral eye control design. To the best of our knowledge, this is the first clinical study conducted at a single center that uses a contralateral eye (or intraindividual), prospective design to assess vulnerabilities specifically in diabetic patients to TB staining.

## 2. Materials and Methods

### 2.1. Patient Population

This prospective, controlled cohort study included 124 eyes (62 patients) with bilateral diabetic retinopathy and visually significant cataracts in which the use of capsule dye was indicated. All patients underwent phacoemulsification, performed by the same surgeon (H. A.), between May 2015 and December 2017.

### 2.2. Inclusion and Exclusion Criteria

Patients with a history of ocular surgery, active ocular inflammation, corneal opacities, pseudoexfoliation syndrome, anterior chamber flare and (or) other signs of possibly altered blood-aqueous barrier, iris neovascularization (rubeosis), uncontrolled glaucoma, congenital cataracts, and traumatic cataracts; those with a history or ongoing chronic use of topical or systemic steroids; or those with poor papillary dilatation (<6 mm) were excluded from the present study.

Patients with a specular microscopy cell density <2000 cell/mm^2^ were excluded. Additionally, any patients who experienced complications from phacoemulsification surgeries, such as posterior capsule rupture or zonular dialysis with vitreous loss, or required any other procedure to dilate the pupil intraoperatively were also excluded.

Each patient's diabetic retinopathy was evaluated by a retina specialist who selected cases with bilateral severe nonproliferative diabetic retinopathy (NPDR) and patients with bilateral high-risk proliferative diabetic retinopathy (PDR). Patients with severe NPDR and high-risk PDR treated with panretinal photocoagulation at least three months prior to cataract surgery were not excluded. However, patients with high-risk PDR that was treated via vitrectomy alone or in combination with other surgical procedures for stabilization of the retina were excluded from this study.

The diagnosis of severe NPDR (4 : 2 : 1 rule of the Early Treatment Diabetic Retinopathy Study (ETDRS) guidelines) was based on any of the following: more than 20 intraretinal hemorrhages in each of 4 quadrants, definite venous beading in 2 + quadrants, prominent intravitreal microvascular abnormalities in 1 + quadrant, and no signs of proliferative diabetic retinopathy. High-risk PDR was defined according to ETDRS guidelines as patients with the following risk factors: (1) presence of neovascularization of the disc (NVD) > one-third of the disc area; (2) NVD with vitreous or preretinal hemorrhage; (3) neovascularization elsewhere ≥ one-half of the disc area with vitreous or preretinal hemorrhage [10].

We excluded diabetic patients with no diabetic retinopathy or mild to moderate grades of NPDR as well as PDR patients with no high-risk characteristics. This decision was based on the need to investigate the advanced stages of the disease as a strong evidence or “marker” for presence of advanced diabetic eye disease with its possible burden on the corneal endothelium. Patients with macular edema and patients treated with intravitreal anti-VEGF were also excluded from this study.

The density of each patient's cataracts was clinically graded by the same surgeon (H. A.) according to the Lens Opacity Classification System (LOCS) III [11], and only cases with soft to moderately hard nuclei were included in the study in an attempt to decrease phacoemulsification energy and procedure time. Patients with hard nuclei were excluded because elimination of these nuclei requires a longer procedure, leading to an increase in the duration of heat liberation, bouncing fragments, and free radical oxygen production. Additionally, the emulsification of hard nuclei requires the use of a large volume of fluids, which can further damage the corneal endothelium and compromise assessment of the effects of use of TB.

Informed consent was obtained from all patients. Ethical approval of this study was provided in accordance with the Declaration of Helsinki by the Medical Research and Ethics Committee of the Assiut University Faculty of Medicine.

### 2.3. Surgical Technique

Randomization was done using an envelope technique wherein, after each patient was placed on the operating bed, the surgeon opened the patient's corresponding envelope, which contained information about whether TB should be used for anterior capsular staining or not. All patients then underwent small incision phacoemulsification using topical anesthesia. A 1% TB solution (Vision Blue®, DORC, Zuidland, Netherlands) was administered via paracentesis at the beginning of the surgery by allowing the aqueous humor to exit the anterior chamber, which became shallow. A resulting pupil block confined the dye to the anterior chamber. The dye was left in the anterior chamber for an average of 5 to 10 seconds, and a dispersive ophthalmic viscosurgical device (OVD) containing a 3.0% sodium hyaluronate and 4.0% chondroitin sulfate (Viscoat, Alcon Inc., Fort Worth, TX) solution was injected to flush out dye-stained aqueous from the anterior chamber (via an intracameral one-step injection) [4], and then, capsulorhexis was conducted. If TB was not used, OVD (Viscoat) was first injected into the anterior chamber via a paracentesis prior to conducting the capsulorhexis.

Phacoemulsification was performed with a 0.9 mm miniflared ABS (aspiration bypass system; Alcon, Inc.) in the torsional mode using a 45° Kelman tip equipped with an ultrasleeve.

An infinity vision phacoemulsification machine with Ozil® torsional technology (Alcon Laboratories Inc., Fort Worth, TX) was used with a stop-and-chop technique for nuclear disassembly. The surgeon was careful to confine all manipulations to the posterior chamber.

A torsional ultrasound amplitude of 100% (0–100%) linear control and intelligent phaco (IP) was used across all cases. IP instructs the machine to provide a short burst of longitudinal power upon sensing occlusion to prevent clogging of the tip. The intraocular lens was placed within the capsular bag in all cases.

Postoperatively, all patients received the same prophylactic regimen consisting of a combination of antibiotic, steroid, and nonsteroidal anti-inflammatory eye drops.

### 2.4. Preoperative, Intraoperative, and Postoperative Evaluation and Measurements

Preoperatively, all patients underwent a complete slit-lamp examination and an indirect ophthalmoscopy to fulfill inclusion and exclusion criteria.

Noncontact specular microcopy (SP-1P; Topcon Corp., Tokyo, Japan) was used to measure baseline endothelial cell density (ECD, endothelial cells/mm^2^), the coefficient of variation (CV, defined as the degree of variation in average corneal endothelial cell surface area, an index of “polymegathism”), the percent of hexagonal cells (% of six-sided cells, an index of “pleomorphism”) [12], and mean central corneal thickness (CCT). The SP-1P specular microscope features a corneal endothelium photography magnification of 254x (on the control panel), with a photography range of 0.25–0.55 mm and a central corneal thickness measurement range of 0.400–0.750 mm. The SP-1P software uses a fully automated analysis method to outline endothelial cell borders and perform calculations. The analyzed image is classified in colors coded according to the number of angles and area of each cell. The cells that are not fully identified are displayed with black boundaries and are excluded from analyzed value calculations. At least 3 endothelial photographs were taken at each time point, and the mean of each parameter was used for comparison. Only captured images with more than 100 analyzable cells per frame were used to obtain maximum accuracy. All micrographs were taken using a central fixation target.

Preoperative and four-week postoperative differences in ECD, CV, % hexagonality, and CCT were obtained for the central cornea of both of each patient's eyes. The percentage of endothelial cell loss was calculated using the following formula (pre = preoperative and post = postoperative):

(1) $$\begin{array}{c} \text{loss of CD}\left(\%\right)=\frac{{\text{CD}}_{\text{pre}}-{\text{CD}}_{\text{post}}}{{\text{CD}}_{\text{pre}}}. \end{array}$$

Some patients presented with an increase in ECD postoperatively, possibly caused by a sampling error resulting in very small preoperative-to-postoperative differences. This group of patients was considered to have no change in their ECD.

Cumulative dissipated energy (CDE) was recorded at the end of each operation from the metrics of the phacoemulsification machine, calculated as (phaco time × average phaco power) + (torsional time × 0.4 × average torsional amplitude), with the factor 0.4 representing the approximate reduction in heat due to dissipation at the incision site as compared to that which occurs with conventional phaco. The endothelial cell analyses were performed, and data were processed in an observer-blinded fashion to prevent observer bias confounding the results.

### 2.5. Statistical Analyses

All descriptive statistical analyses were performed using SPSS software (version 19.0, IBM Corp., Armonk, NY, USA). Patient characteristics were summarized as means, standard deviations (SD), and minimums and maximums for continuous variables and frequencies and percentages for categorical variables. Student's *t*-test was used to compare all sample means. A *P* value of less than 0.05 was considered to be statistically significant. A correlation between endothelial cell loss (CD loss (%)) and CDE was assessed using the Pearson correlation coefficient (*r*). The sample size was chosen to achieve a statistical power of greater than 80% for the group comparison based on a two-sample Wilcoxon rank-sum test at a 5% significance level.

## 3. Results

Per our study protocol's inclusion and exclusion criteria, a final 65 patients (130 eyes) were recruited. Two patients were excluded due to missing follow-up visits four weeks postoperatively, and one patient was excluded due to a posterior capsule rupture and subsequent vitreous loss during phacoemulsification. Given this, the final sample size included 124 eyes (62 patients: 26 males (42%) and 36 females (58%)) with a mean age of 50.80 (range: 28 to 63) and a mean duration of diabetes mellitus of 16.36 years (range: 9 to 33).

The mean (±SD) CDE was 2.49 ± 1.82 in the study eyes (minimum: 0.00, maximum: 8.11) and 2.59 ± 1.75 in the control eyes (minimum: 0.00, maximum: 7.78). There was no significant difference in CDE between the two groups (*P*=0.273). Four weeks postoperatively, there was also no significant difference in ECD between the two groups (Table 1).

The degree of endothelial cell loss (CD loss%) four weeks postoperatively was positively correlated with CDE in both the study and control eyes (*r* = 0.233 and 0.355, respectively). The mean (±SD) CD loss% four weeks postoperatively was 7.23 ± 13.31 for the study eyes and 9.94 ± 9.36 for the control eyes. There was no significant difference in CD loss% between the groups (*P*=0.157).

Univariate analyses showed no significant differences in the percentage of hexagonal cells or CVs of study and control eyes at either the preoperative or four-week postoperative time points (Table 1).

Mean CCT was significantly increased at the four-week follow-up visit compared to preoperative CCTs in both the study and control eyes (*P*=0.0002 and 0.031, respectively). However, CCT was not significantly different between the groups (Table 1).

## 4. Discussion

In the present study, we assessed the safety of TB in terms of corneal endothelium function and ultrastructure four weeks after its use in anterior capsule staining during phacoemulsification in patients with diabetic retinopathy. One eye was treated with TB (study eye) while the contralateral eye was not (control eye).

We observed no significant differences in ECD, % hexagonality, CV, or CCT between the two groups, indicating that TB is not significantly toxic to the relatively fragile corneal endothelial cells of patients with diabetic retinopathy.

It is well known that the corneal endothelium in diabetic patients is relatively fragile and thus more vulnerable to damage caused by surgical trauma than the corneal endothelium of healthy individuals. This damage is reflected by changed ECD, % hexagonality, and CV values. This loss and morphological abnormality is also often coupled with increased CCT [9, 13, 14]. Van Dooren et al. have demonstrated the low toxicity of TB to corneal endothelial cells during cataract surgery [6]. However, this has not been assessed specifically in a diabetic population, making our study particularly novel.

To detect any potential toxic effect of TB on the vulnerable endothelium of diabetic corneas during phacoemulsification, we attempted to address and minimize all possible operative confounders that have a proven damaging effect on corneal endothelium during phacoemulsification [15, 16]. Furthermore, the present study utilized intraindividual controls (the contralateral eye) to normalize any individualized biological responses. Our selection of only those cataracts with soft or moderately hard nuclei and use of torsional ultrasound energy effectively allowed for the reduction of CDE. Additionally, our use of the same nuclear disassembly technique (stop-and-chop) by the same surgeon allowed for uniformity across all patients and operative manipulations. Finally, our use of the soft-shell technique allowed for protection of the corneal endothelium.

At present, corneal endothelial injury associated with phacoemulsification is typically assessed via specular microscopy and described in terms of changes to cell density or morphology. The results of the present study clearly demonstrated no significant difference between study and control eyes in terms of CDE, % hexagonality, CV, or CCT values with or without the use of TB. This result is consistent with those of previous studies conducted exclusively on nondiabetic patients [1, 6]. However, in these studies, the anterior capsule was stained under air and as such the investigators were unable to assess only the influence of the dye on the corneal endothelium [17]. Sharma and Panwar [18] injected TB directly into the capsular bag during posterior capsule staining for cataract surgery and reported no significant corneal endothelial damage as a result. In the present study, we injected TB directly into the anterior chamber and irrigated the corneal endothelium with the dye, thereby potentially influencing cell viability. However, we found no significant toxicity to the vulnerable endothelium of diabetic patients, indicating that this dye can be safely injected into the anterior chamber of diabetic patients.

The doses used here and elsewhere are an additional factor which warrants exploration. In a previous study, the endothelium of human donor corneas was stained for one minute with 0.3% TB without any indication of endothelial cell loss [19].

Both the present study and work by Chung et al. [1] used a TB concentration of 1%, while Nagashima et al. [20] used a lower concentration (0.06%). All found no significant difference in corneal endothelium changes between study and control eyes. Despite these important findings, the above studies did not investigate the impact of TB staining specifically on diabetic corneal endothelium as we have here, rendering our study particularly novel.

Our study had some limitations which warrant discussion. First, we did not measure changes in ECD and CCT the day after surgery. Second, we conducted only one postoperative follow-up at four weeks. This limited our ability to trace endothelial changes across a longer time scale to assess for recovery of hexagonality of endothelial cells in diabetic corneas, for example, which has been found to take more than three months [21]. However, we intentionally designed the study with this relatively short follow-up interval, taking into consideration that the intraindividual nature of the observations with one study eye and a control (contralateral) eye of the same patient who has diabetic retinopathy and undergone phacoemulsification would risk the possibility of losing many patients by exclusion, should they have needed an intravitreal anti-VEGF injection shortly after the operation. Such treatment in either eye before the follow-up visit could have theoretically confounded the corneal endothelium status. Furthermore, our sample size was small, so larger cohorts should be used in future studies. Further studies on hard cataract are also recommended.

In conclusion, direct intracameral administration of TB during cataract surgery for staining of the anterior capsule did not result in any significant corneal endothelial changes on specular microscopy in patients with severe NPDR or high-risk PDR at 4 weeks postoperatively, in spite of the increased vulnerability of corneal endothelial cells in these diabetic patients.

## Figures and Tables

**Table 1:** Corneal endothelial cell density (cells/mm^2^), corneal endothelial cell hexagonality (%), coefficient of variation of cell size, and central corneal thickness (*μ*m) preoperatively and 4 weeks postoperatively.

Exam time	Measured item	Study eyes (TB used)		Control eyes (TB not used)		95% CI for mean difference	*P* ^*∗*^
		Mean	SD	Mean	SD		
Preoperative	ECD	2706.85	297.46	2753.48	453.53	−22.22, 115.47	0.181
	Hex %	49.2	6.06	48.59	5.41	−1.66, 0.41	0.233
	CV	35.90	5.50	36.00	5.61	−0.65, 0.85	0.793
	CCT	500.59	30.71	499.90	31.40	−3.98, 2.60	0.677

4 weeks postoperative	ECD	2506.74	413.99	2466.34	369.12	−141.31, 60.52	0.427
	Hex %	45.33	5.99	45.63	5.04	−1.07, 1.34	0.952
	CV	38.16	6.15	38.64	5.72	−0.81, 1.80	0.464
	CCT	504.57	30.22	505.69	33.74	−4.67, 6.89	0.386

^*∗*^Paired Student's *t*-test. ECD = corneal endothelial cell density (cells/mm^2^); Hex % = corneal endothelial cell hexagonality (%); CV = coefficient of variation of cell size; CCT = central corneal thickness (*μ*m).

## Data Availability

No data were used to support this study.

## Abbreviations

CCT: Central corneal thickness
CDE: Cumulative dissipated energy
CV: Coefficient of variation
ECD: Endothelial cell density
NPDR: Nonproliferative diabetic retinopathy
OVD: Ophthalmic viscosurgical device
PDR: Proliferative diabetic retinopathy
SD: Standard deviation
TB: Trypan blue.

## References

[1] Chung C. F., Liang C. C., Lai J. S., Lo E. S., Lam D. S. (2005). Safety of trypan blue 1% and indocyanine green 0.5% in assisting visualization of anterior capsule during phacoemulsification in mature cataract. *Journal of Cataract and Refractive Surgery*.

[2] Dada V. K., Sharma N., Sudan R., Sethi H., Dada T., Pangtey M. S. (2004). Anterior capsule staining for capsulorhexis in cases of white cataract. *Journal of Cataract and Refractive Surgery*.

[3] Jacob S., Agarwal A., Agarwal A. (2002). Trypan blue as an adjunct for safe phacoemulsification in eyes with white cataract. *Journal of Cataract and Refractive Surgery*.

[4] Jhanji V., Chan E., Das S., Zhang H., Vajpayee R. B. (2011). Trypan blue dye for anterior segment surgeries. *Eye*.

[5] Melles G. R. J., de Waard P. W. T., Pameyer J. H., Houdijn Beekhuis W. (1999). Trypan blue capsule staining to visualizethe capsulorhexis in cataract surgery. *Journal of Cataract and Refractive Surgery*.

[6] van Dooren B. T. H., de Waard P. W. T., Poort-van Nouhuys H., Beekhuis W. H., Melles G. R. J. (2002). Corneal endothelial cell density after trypan blue capsule staining in cataract surgery. *Journal of Cataract and Refractive Surgery*.

[7] Lutty G. A. (2013). Effects of diabetes on the eye. *Investigative Opthalmology and Visual Science*.

[8] Srinivas S. P. (2010). Dynamic regulation of barrier integrity of the corneal endothelium. *Optometry and Vision Science*.

[9] Li M. H., Fu X. L., Yang W. F. (2016). Effect and risk factors for corneal endothelial cells after phacoemulsification in diabetic cataract patients. *International Eye Science*.

[10] Early Treatment Diabetic Retinopathy Study Research Group (1991). Grading diabetic retinopathy from stereoscopic color fundus photographs—an extension of the modified Airlie House classification. *Ophthalmology*.

[11] Chylack L. T., Wolfe J. K., Singer D. M (1993). The lens opacities classification system III. The longitudinal study of cataract study group. *Archives of ophthalmology (Chicago, III: 1960)*.

[12] Bonnell A. J., Cymbor M. (2012). Under the specular microscope: take a closer look at the clinical and financial benefits that non- contact specular microscopy could offer your practice. *Review of optometry*.

[13] Lee J.-S., Lee J.-E., Choi H.-Y., Oum B.-S., Cho B. M. (2005). Corneal endothelial cell change after phacoemulsification relative to the severity of diabetic retinopathy. *Journal of Cataract and Refractive Surgery*.

[14] Misra S. L., Goh Y. W., Patel D. V., Riley A. F., McGhee C. N. J. (2015). Corneal microstructural changes in nerve fiber, endothelial and epithelial density after cataract surgery in patients with diabetes mellitus. *Cornea*.

[15] Sorrentino F. S., Bonifazzi C., Parmeggiani F., Perri P. (2016). A pilot study to propose a “harm scale,” a new method to predict risk of harm to the corneal endothelium caused by longitudinal phacoemulsification, and the subsequent effect of endothelial damage on post operative visual acuity. *PLoS One*.

[16] Takahashi H. (2016). Corneal endothelium and phacoemulsification. *Cornea*.

[17] Wong V. W. Y., Lai T. Y. Y., Lee G. K. Y., Lam P. T. H., Lam D. S. C. (2006). A prospective study on trypan blue capsule staining under air vs under viscoelastic. *Eye*.

[18] Sharma P., Panwar M. (2013). Trypan blue injection into the capsular bag during phacoemulsification: initial postoperative posterior capsule opacification results. *Journal of Cataract and Refractive Surgery*.

[19] Sperling S. (1986). Evaluation of the endothelium of human donor corneas by induced dilation of intercellular spaces and trypan blue. *Graefe’s Archive for Clinical and Experimental Ophthalmology*.

[20] Nagashima T., Yuda K., Hayashi T. (2019). Comparison of trypan blue and Brilliant Blue G for staining of the anterior lens capsule during cataract surgery: short-term results. *International Ophthalmology*.

[21] Tang Y., Chen X., Zhang X., Tang Q., Liu S., Yao K. (2017). Clinical evaluation of corneal changes after phacoemulsification in diabetic and non-diabetic cataract patients, a systematic review and meta-analysis. *Scientific Reports*.

